# Genetic diversity and molecular epidemiology of Middle East Respiratory Syndrome Coronavirus in dromedaries in Ethiopia, 2017–2020

**DOI:** 10.1080/22221751.2022.2164218

**Published:** 2023-01-27

**Authors:** Ziqi Zhou, Abraham Ali, Elias Walelign, Getnet F. Demissie, Ihab El Masry, Takele Abayneh, Belayneh Getachew, Pavithra Krishnan, Daisy Y.M. Ng, Emma Gardner, Yilma Makonnen, Eve Miguel, Véronique Chevalier, Daniel K. Chu, Ray T. Y. So, Sophie Von Dobschuetz, Gezahegne Mamo, Leo L. M. Poon, Malik Peiris

**Affiliations:** a School of Public Health, The University of Hong Kong, Hong Kong Special Administrative Region, People’s Republic of China; b Bacterial, Parasitic and Zoonotic Diseases Research Directorate, Ethiopian Public Health Institute, Addis Ababa, Ethiopia; c Department of Veterinary Microbiology, Immunology and Public Health, College of Veterinary Medicine and Agriculture, Addis Ababa University, Bishoftu, Ethiopia; d Food and Agriculture Organization, Emergency Centre for Transboundary Animal Diseases, Addis Ababa, Ethiopia; e College of Veterinary Medicine, Department of Veterinary Epidemiology, Microbiology and Public Health, Haramaya University, Haramaya, Ethiopia; f Food and Agriculture Organization of the United Nations, Rome, Italy; g National Veterinary Institute, Debre Zeit, Ethiopia; h Food and Agriculture Organization, Subregional Office for Eastern Africa, Addis Ababa, Ethiopia; i Animal, Santé, Territoires, Risques et Ecosystèmes, Centre de Coopération Internationale en Recherche Agronomique pour le Développement, Institut National de la Recherche Agronomique, Université de Montpellier, Montpellier, France; j Maladies Infectieuses et Vecteurs: Ecologie Genetique, Evolution et Controle, L’Institut de Recherche pour le Developpment, CNRS, Montpellier, France; k International Center of Research in Agriculture for Development (CIRAD), UMR ASTRE, Montpellier, France; l CIRAD, UMR ASTRE, Antananarivo, Madagascar; m Epidemiology and Clinical Research Unit, Institut Pasteur de Madagascar, Antananarivo, Madagascar; n UK Health Security Agency, London, UK

**Keywords:** MERS-CoV, Ethiopia, genetic instability, molecular epidemiology, evolution

## Abstract

Middle East respiratory syndrome coronavirus (MERS-CoV) is enzootic in dromedary camels and causes zoonotic infection and disease in humans. Although over 80% of the global population of infected dromedary camels are found in Africa, zoonotic disease had only been reported in the Arabia Peninsula and travel-associated disease has been reported elsewhere. In this study, genetic diversity and molecular epidemiology of MERS-CoV in dromedary camels in Ethiopia were investigated during 2017–2020. Of 1766 nasal swab samples collected, 61 (3.5%) were detected positive for MERS-CoV RNA. Of 484 turbinate swab samples collected, 10 (2.1%) were detected positive for MERS-CoV RNA. Twenty-five whole genome sequences were obtained from these MERS-CoV positive samples. Phylogenetically, these Ethiopian camel-originated MERS-CoV belonged to clade C2, clustering with other East African camel strains. Virus sequences from camel herds clustered geographically while in an abattoir, two distinct phylogenetic clusters of MERS-CoVs were observed in two sequential sampling collections, which indicates the greater genetic diversity of MERS-CoV in abattoirs. In contrast to clade A and B viruses from the Arabian Peninsula, clade C camel-originated MERS-CoV from Ethiopia had various nucleotide insertions and deletions in non-structural gene nsp3, accessory genes ORF3 and ORF5 and structural gene N. This study demonstrates the genetic instability of MERS-CoV in dromedaries in East Africa, which indicates that the virus is still actively adapting to its camel host. The impact of the observed nucleotide insertions and deletions on virus evolution, viral fitness, and zoonotic potential deserves further study.

## Introduction

The Middle East respiratory syndrome coronavirus (MERS-CoV) was first identified in a patient with the severe respiratory syndrome in Saudi Arabia in 2012 [1]. As of June 2022, 2591 laboratory-confirmed cases were reported to the World Health Organization with a fatality rate of 34.5% [2]. It remains a cause for global public health concern.

Dromedary camels are the source of zoonotic MERS-CoV infection [1,3]. According to the Food and Agriculture Organization (FAO), the number of dromedary camels worldwide is estimated to be 39 million in 2020, of which over 80% are in Africa [4]. In East Africa, where there is the highest density of dromedary camels in the world, Ethiopia, Sudan, Somalia and Kenya have 1.6, 4.9, 7.3 and 4.7 million camels, respectively, accounting for almost 50% of the global population [4]. MERS-CoV is highly prevalent in dromedary camels, in both Africa and the Arabian Peninsula [5]. The earliest evidence of MERS-CoV infection in dromedaries dates back to 1983, based on the retrospective detection of MERS-CoV antibodies in camel sera collected in Sudan and Somalia [6]. In more recent studies in Egypt, Kenya, Ethiopia, and Somalia, over 90% of camels were seropositive to MERS-CoV and nasal viral RNA positive rate by RT–PCR ranged from 0.23% to 15.4% [5,7–9].

Despite the high prevalence of MERS-CoV in camels, zoonotic human disease has only been reported in the Arabia Peninsula although travel-associated outbreaks have been reported elsewhere [10]. Zoonotic infection has also sometimes caused transmission among humans with outbreaks of more than 100 individuals, which have repeatedly occurred in healthcare settings in the Arabian Peninsula and once in the Republic of Korea [11,12]. The apparent absence of zoonotic MERS disease from Africa remains an enigma. Previous studies showed that MERS-CoV circulating in Arabian Peninsula and Africa exhibit region-dependent genetic diversity and differ in their phenotypic traits [13,14]. Genetically distinct clade C MERS-CoV from Africa (Ethiopia, Kenya, Burkina Faso, and Nigeria) had lower replication competence in *ex vivo* cultures of the human bronchus and lungs in comparison with clade A and B strains from Saudi Arabia, which may possibly indicate the reduced human pathogenic potential of African MERS-CoV viruses [14]. It has been shown that deletion in the ORF4b gene and amino acid differences in the spike protein observed in clade C viruses may contribute to this phenotype difference [13,14]. While MERS-CoV antibodies have been detected in humans with exposure to camels in the Arabian Peninsula, with some studies showing up to 50% of camel workers (e.g. slaughterhouse workers and herders) to be seropositive, such evidence was less often reported from Africa, and if so, only at very low prevalence [15–19]. However, virologically confirmed infection is not always associated with antibody responses [20] and sero-epidemiology may markedly underestimate human infection in Africa. The recent detection of MERS-CoV specific T cell responses in around 30% of camel workers in an abattoir in Kano, Nigeria, despite an absence of detectable antibody to MERS-CoV, suggests that infection of camel-exposed populations may not be uncommon [21]. In a cohort of 262 camel handlers in Kenya followed longitudinally for two years, where camels were detected to be MERS-CoV positive, three camel handlers were detected MERS-CoV RNA positive [22,23]. Given the high prevalence and widespread presence of MERS-CoV infection in dromedary camels in Africa, there is a need for continued surveillance and studies at the camel-human interface in Africa in order to better define virus genetic diversity, evolution and phenotypic characterization of MERS-CoV circulating in Africa. This would be beneficial for understanding zoonotic risks, introducing risk-reduction measures and developing countermeasures against MERS-CoV in camel populations and humans. This study reports the genetic characteristics and molecular epidemiology of MERS-CoV in camels in Ethiopia.

## Material and methods

### Sample collection

1766 nasal swabs were collected from January 2017 to January 2020, and 484 turbinate swabs were collected from September 2019 to September 2020, from dromedary camels from camel herds and slaughterhouses in different regions of Ethiopia. Slaughterhouses sampled were located in Akaki and Babile. The Akaki slaughterhouse sourced camels from diverse regions in Ethiopia, predominantly the Borna area (Oromia region), Menze (Amahar region bordering Afar), from Afar (Afar region) and from the Methara area (Oromia Region). The number of camels slaughtered depends on demand and was around 3–10 camels per day. Camels brought to the abattoir stayed for days, ranging from a few days to a few weeks. The camels were kept in a single pen hence camels from different areas were mixed together. The slaughterhouse in Babile sourced camels from the Gursum, Babile and Somale regions nearby and camels were held for 3 or more days prior to slaughter. Camels were housed in pens where animals from different sources were held together and approximately 3–5 camels were slaughtered daily, depending on demand. In general, camels in slaughterhouse were apparently healthy and didn’t show any clinical symptoms. Ante and post-mortem inspections of the camels slaughtered at the abattoir was carried out by veterinary inspectors assigned by the Addis Ababa Abattoirs enterprise. Camels with clinical signs of disease would not be accepted for slaughter at the abattoir.

After collection, swabs were put into virus transport medium and placed in cool boxes with ice packs for transport. On arrival at the local laboratories, samples were stored at −80 °C. When shipped to Hong Kong University (HKU) laboratory, samples were frozen in dry ice and then stored at −80 °C upon arrival, until testing.

### MERS-CoV detection

Total nucleic acid was extracted from the samples using the automated MagPure 96 (Roche, Basel, Switzerland) or NucliSENS easyMAG 24 system (bioMérieux, Craponne, France). In the MagPure 96 system, viral RNA was extracted using the Viral RNA Small volume kit (Roche, Basel, Switzerland) by adding 200μl of the virus transport medium and eluting in 50μl H2O. In the NucliSENS easyMAG 24 system, viral RNA was extracted using 200μl of the virus transport medium and eluted in 60μl of elution buffer. The MERS-CoV RT-qPCR diagnostic tests were performed according to the WHO guideline [24]. A screening assay targeting the upE gene was first performed. Positive samples were confirmed by a second RT-qPCR assay targeting the ORF1a gene [25].

### MERS-CoV whole genome sequencing

MERS-CoV whole genome sequencing was performed as previously described [26]. MERS-CoV cDNAs of different regions of the viral genome were synthesized using multiple gene-specific primers with the SuperScript IV First-Strand Synthesis System, according to the manufacture’s recommendations (Thermofisher, Massachusetts, US). Nested PCRs with primers to produce overlapping DNA fragments were performed to amplify the whole genome using the TaKaRa LA Taq kit (Takara, Shiga, Japan). After purification using the QIAquick PCR Purification Kit (QIAGEN, Hilden, Germany), PCR products were sent to the HKU Centre for PanorOmic Sciences (CPOS) for NGS sequencing. The library was prepared by The Nextera XT DNA Library Preparation Kit (Illumina, California, US), followed by sequencing on the Illumina NovaSeq 6000 platform. Genome consensus sequences were generated through mapping to a reference MERS-CoV genome, the human strain EMC/2012 (Genbank: JX869059.2) using the BWA software, with a minimum coverage of 100 reads. Indels in nsp3, ORF3, ORF5 and N genes detected in the NGS sequencing were further confirmed by Sanger sequencing.

### Phylogenetic analysis

Representative sequences (*n* = 75) in clades A, B and C of MERS-CoV (Table S1) and a sequence of MERS-CoV-related bat coronavirus (Genbank: KC869678.4) were downloaded from Genbank (All clade C whole genome sequences available in Genbank were included). These representative sequences and 25 whole genome MERS-CoV sequences from Ethiopian camels were first aligned using MAFFT version 6 software and phylogenetic analysis was done using the IQ-TREE (v.2.1.3) employing the GTR + F + R4 nucleotide substitution model (best-fit model searched by IQ-TREE) with the MERS-CoV-related bat coronavirus sequence as outgroup.

## Results

### Phylogenetic analysis of MERS-CoV in Ethiopia

**Figure 1.**
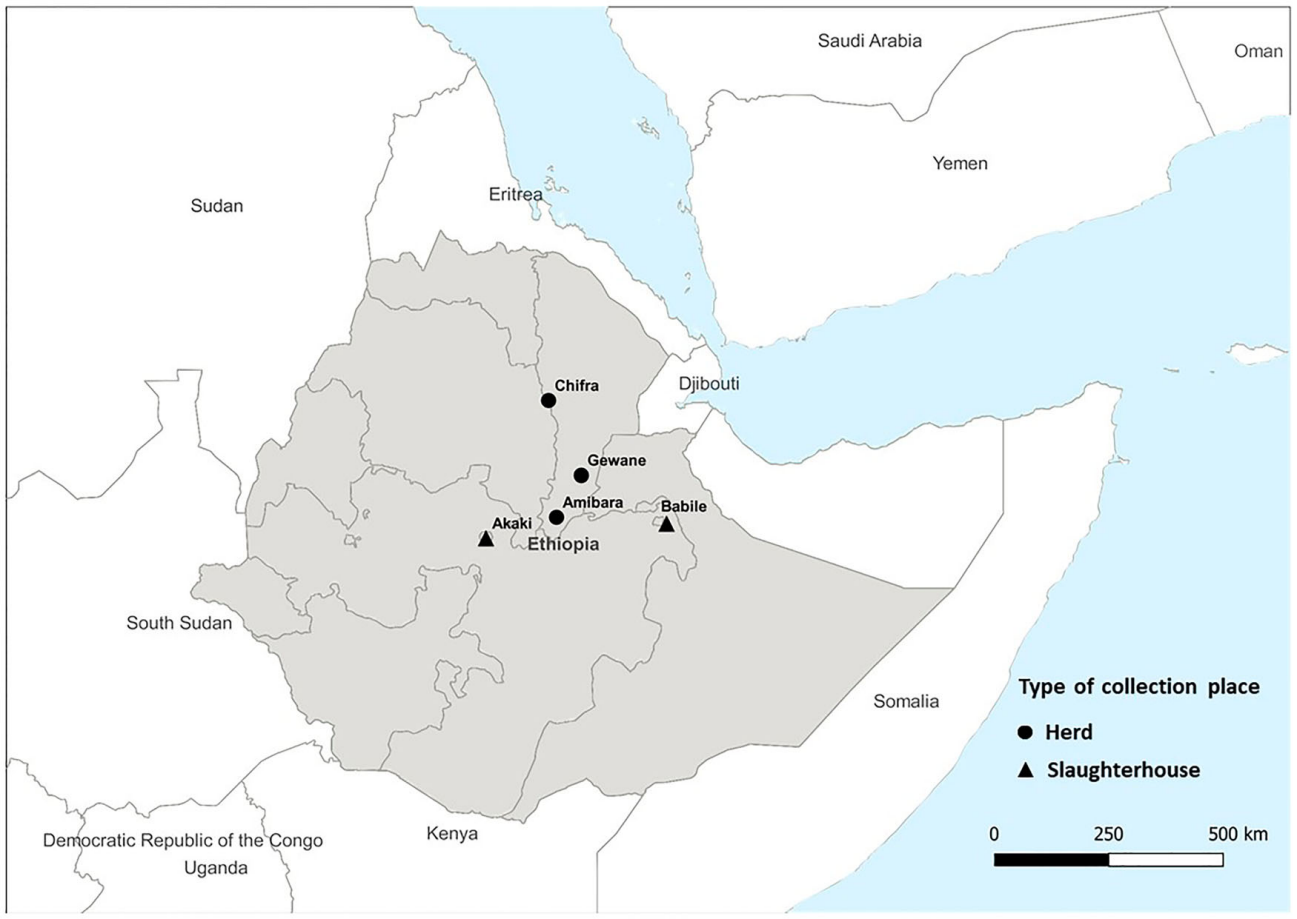
The location and type of collection site of the samples with whole genome sequences. Samples with whole genome sequences were collected in Amibara (*n* = 11), Gewane (*n* = 3), and Chifra (*n* = 3) from Afar region, Babile (*n* = 3) from Oromia region, and Akaki (*n* = 5) in the capital city, Addis Ababa.

**Figure 2.**
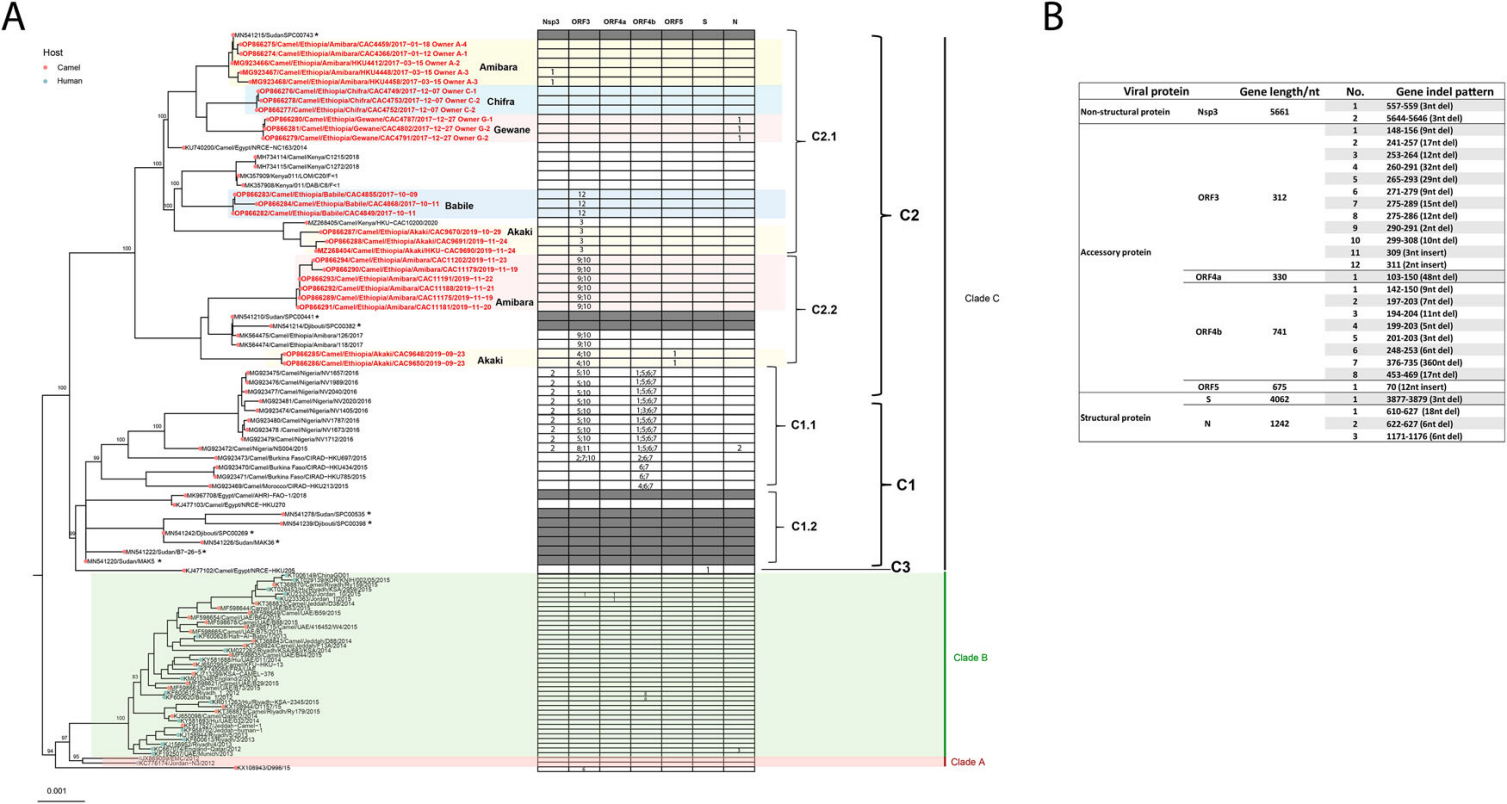
(A) Phylogenetic analysis of MERS-CoV whole genome sequences obtained in this study using IQ-TREE. The tree is rooted against MERS-CoV-related bat coronavirus Coronavirus Neoromicia/PML-PHE1/RSA/2011(GenBank: KC869678.4), which was removed from the tree due to the long branch length. Selective bootstrap values are shown. The MERS-CoV clades designations are denoted based on the previous studies [28,47]. Whole genome sequences labelled in red are from this study and those in black are sequences downloaded from Genbank. S gene partial sequences from the previous study with the length of 1046 bp are labelled with asterisk [28]. Indel patterns of nsp3, ORF3, ORF4a, ORF4b, ORF5, S, and N genes observed in the sequences are shown in the table within the tree (S gene partial sequences and sequence MK967708/Egypt/Camel/AHRI−FAO−1/2018 are excluded). Two clade B Saudi Arabian viruses that show deletions in ORF4b are included for comparison. The sampling places (Amibara, Chifra, Gewane, Babile, Akaki) of the sequences are labelled in the tree. (B) Details of the indel patterns of the sequences in the phylogenetic trees.

Of 1766 nasal swab samples collected from January 2017 to January 2020, 61 (3.45%) were MERS-CoV RNA positive. Of 484 turbinate swab samples collected from September 2019 to September 2020, 10 (2.07%) were detected positive. Twenty-five whole genome sequences of MERS-CoVs were obtained from these samples (sample information listed in Table S2). The location and type of collection site of the sequences obtained are indicated in the Figure 1. Phylogenetic analysis was done using the IQ-TREE with a MERS-CoV-related bat coronavirus (GenBank: KC869678.4) as an outgroup [27]. In the phylogenetic tree, our result supports the previous classification that all MERS-CoV from Africa are clade C viruses. Within clade C1, clade C1.1 viruses were West African strains from Burkina Faso, Nigeria and Morocco, and C1.2 are East African strains from Sudan, Djibouti, and Egypt. In this study, clade C2 was further divided into two novel subclades designated as C2.1 and C2.2. The newly sequenced Ethiopian camel-originated MERS-CoV fall within clades C2.1 and C2.2, along with other East African camel-originated strains from Sudan, Djibouti, Kenya, and Egypt (Figure 2(A)). A highly distinct Egyptian strain is designated as clade C3 based on a previous study [28].

### Molecular epidemiology

Interestingly, of virus sequences collected from herds in 2017, those Gewane (CAC4787, CAC4791, CAC4802), Chifra (CAC4749, CAC4752, CAC4753) and Amibara (CAC4366, HKU4412, HKU4448, HKU4458, CAC4459) clustered within each region and were phylogenetically separate from virus sequences from the Babile slaughterhouse (CAC4849, CAC4855, CAC4868). Virus sequences from camels sampled from herds owned by different owners in the same district clustered together. However, in Amibara, viruses sampled in 2017 and 2019 from this study, and viruses sampled in 2017 from the other study (Genbank: MK564475 and MK564474) clustered separately [29]. Nonetheless, these sequences from Amibara did not cluster together with sequences from other districts.

Among the sequences collected from a local slaughterhouse in Akaki from September to November 2019, viruses CAC9648 and CAC9650 clustered together, falling into subclade C2.2. However, viruses (CAC9670, CAC9690, CAC9691) obtained from this same slaughterhouse in October (*n* = 1) or November (*n *= 2) 2019 clustered separately in clade C2.1 and were markedly different (over 250 nucleotide differences) compared to CAC9648 and CAC9650. Nor were these viruses within each subclade identical to each other. This finding was explained by the fact that slaughterhouses receive camels from geographically diverse areas, thus explaining genetically diverse viruses being detected. In another local slaughterhouse in Babile, viruses (CAC4849, CAC4855, CAC4868) from camels with different origins collected in Oct 2017 clustered together, which indicate the possible virus cross-transmission occurred within the slaughterhouse (Table S2).

### Genetic characteristics of MERS-CoV in Ethiopia

Of the total 35 whole genome sequences currently available from East Africa (Egypt = 3; Kenya = 5; Ethiopia = 27) from this and previous studies, 23 (66.7%) virus sequences harboured indels (partial sequences and sequence MK967708/Egypt/Camel/AHRI−FAO−1/2018 without peer review are excluded). Of the 25 whole genome sequences obtained in Ethiopia in the present study, 19 (76%) sequences harboured indels. These indels were observed in nsp3, ORF3, ORF5 and N gene (Table S1). Unlike East African MERS-CoV, all the whole genome sequences (*n* = 13) from West Africa available in Genbank (Nigeria = 9; Burkina Faso = 3; Morocco = 1) harboured indels, mainly in ORF3 and ORF4b [30].

**Figure 3.**
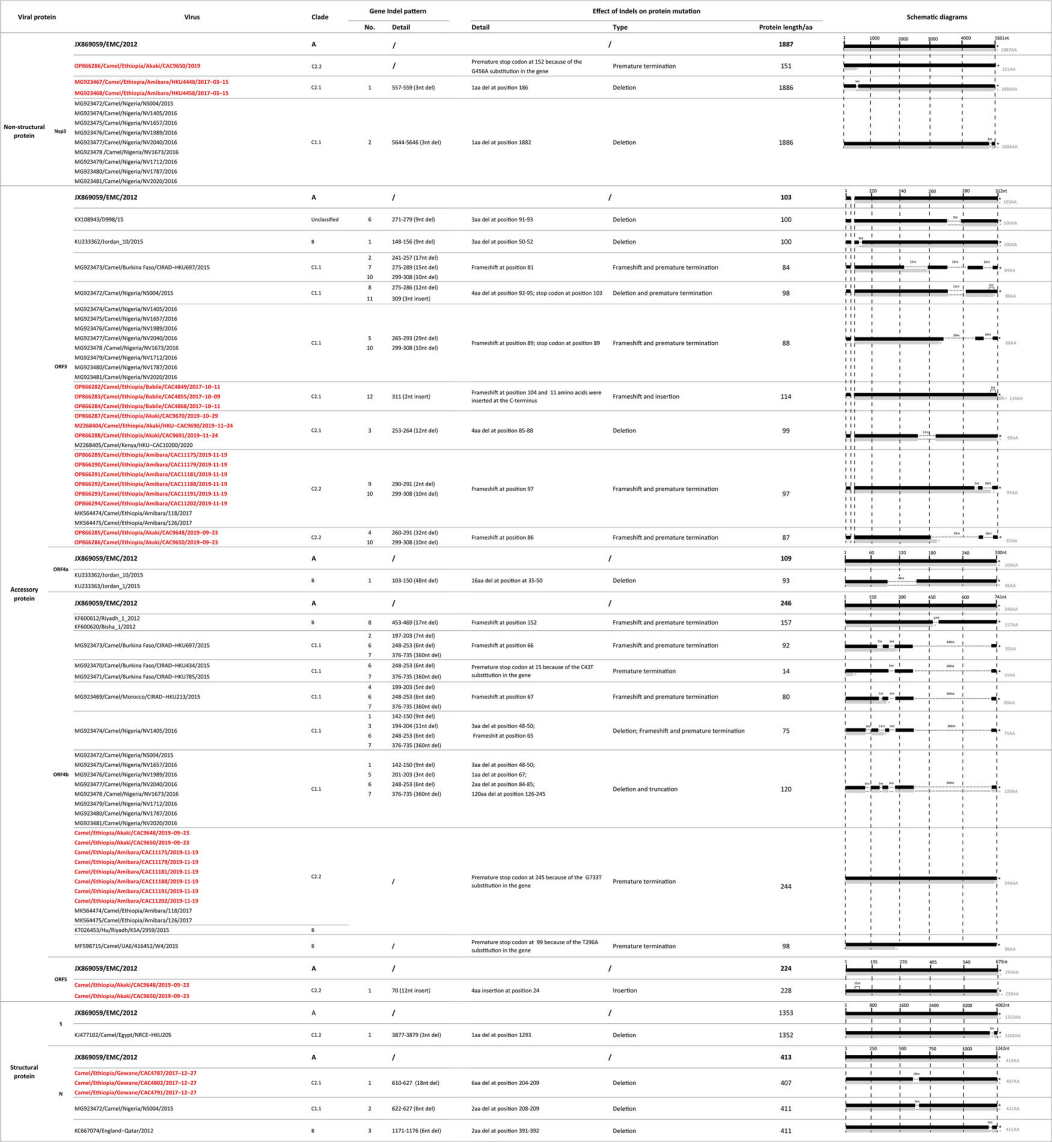
Schematic of the indels and their impact on encoded proteins. A clade A prototype virus, JX869059/EMC/2012 served as reference.

It was previously reported that clade C1.1 viruses from Burkina Faso and Nigeria all harboured large ORF4b gene deletions, which contributed to a reduction of replication competence in human respiratory cells [13]. Viruses from our study in Ethiopia did not have ORF4b gene deletions. However, a substitution at the position of G733 T in ORF4b gene in viruses (CAC9648, CAC9650, CAC11175, CAC11179, CAC11181, CAC11188, CAC11191 and CAC11202) led to a premature stop codon, deleting 2 amino acids at the C-terminus of the ORF4b protein (Figures 2 and 3).

Multiple ORF3 indel patterns were observed in our sequences, similar to other clade C1 viruses. The patterns are geographic subclade specific. For example, viruses from Babile (CAC4855, CAC4868 and CAC4849) contained 2nt insertion at the C-terminus of ORF3, causing the frameshift and predicted to extend ORF3 protein with 11 more amino acids. Among 2 subclade viruses from Akaki, CAC9648 and CAC9650 had Pattern 4 and 10 deletions (42nt in total), while the other three (CAC9670, CAC9690 and CAC9691), have Pattern 3 deletion (12nt) in ORF3 gene. In clade C1.1 viruses, Pattern 5 and 10 deletions were found in most of the Nigerian viruses resulting in 15 amino acid deletions. Notably, KX108943/D998/15 from UAE and a clade B virus KU233362/Jordan_10/2015 from Jordan contained a 9nt deletion in ORF3 gene, resulting in 3 amino acid deletions (Figure 3).

Indels of nsp3, ORF5 and N protein were also observed in the sequences obtained from this study but were less frequent than ORF3 changes. In nsp3 gene, clade C2.1 virus HKU4448 and HKU4458 collected from Amibara in 2017 contained a 3nt deletion from position 557–559 resulting in a 1 amino acid deletion. Viruses from Nigeria in the same subclade of Clade C 1.1 also contained 3nt deletions in nsp3 gene resulting in a 1 amino acid deletion but in a different position. In contrast, a substitution at the position G456A in clade C2.2 virus CAC9650 collected from Akaki slaughterhouse in 2019 led to a premature stop codon and the nsp3 protein was predicted to be 151aa in length, much shorter than that of the reference strain JX869059/EMC/2012 (1887aa in length). Interestingly, viruses CAC9648 and CAC9650 had 4 amino acid insertions in ORF5 protein. In N gene, there were 18nt deletions in sequences collected from Gewane in 2017, predicted to be a 9 amino acid deletion in the protein encoded. N protein deletions were also found in a clade C1.1 virus (NS004) from Nigeria and a clade B virus (KC667074/England−Qatar/2012) (Figure 3).

Nucleotide insertions and deletions were rare in viruses from the Arabian Peninsula. Large ORF4a deletions (48nt) were found in two clade B sequences from a hospital outbreak in Jordan in 2015, but these were different to deletions observed so far from clade C in Africa [31]. While the gene deletions observed in West (Nigeria, Burkina Faso) and North (Morocco) Africa appear to be related (e.g. ORF4b deletion of nucleotides 248-253) with deletions increasing progressively, those in East Africa appear diverse and independent. There was only one example of a deletion (N gene nucleotides 610-627) common between West (MG923472/camel/Nigeria/NS004/2015) and East Africa (OP866280/Camel/Ethiopia/Gewane/CAC4787/2017-12-27).

## Discussion

Ethiopia has an estimated 1.6 million dromedary camels and is an important source of camels for trade to Egypt and the Arabian Peninsula through Djibouti and Somalia [32]. With evidence of human infection found in camel-exposed populations in Africa, it is critical to conduct MERS-CoV surveillance studies to understand the risk of zoonotic infection and monitor ongoing virus evolution. In the present study, we investigated MERS-CoV genetic diversity and molecular epidemiology of MERS-CoV in camels in Ethiopia.

Phylogenetic analysis of whole genome sequences obtained from this study showed that MERS-CoVs from Ethiopia belong to clade C2.1 and C2.2 within the African clade C, clustering with other East African strains. Within Ethiopia, MERS-CoV sequences of 2017 strains clustered geographically by location of sample collection, e.g. those from Amibara, Chifra, and Gewane (Figure 2). This is plausible because camel herders from the same clans in the same districts may graze camels in the same area where there were abundant feed and water sources, thus facilitating transmission of the viruses within and among herds through direct or indirect contact. Even though there were three different phylogenetic clusters of virus sequences from Amibara, these sequences formed separate subclades, genetically different to those from other districts. It is likely that our sampling was not representative of the whole area of the Amibara district and there may be more diverse MERS-CoV circulating in the area. Across the whole virus genome sequenced (around 30 kb), viruses within the same district had 0–25 nucleotide differences while the nucleotide differences among different districts was greater, ranging from 62 to 275.

However, virus sequences obtained from the camel slaughterhouse in Akaki showed greater genetic diversity (from 4 to 262 nucleotide differences across the approx. 30 kb genome sequenced) reflecting the fact that camels of different sources were held and slaughtered in slaughterhouses. Dromedary camels experimentally infected with MERS-CoV started to shed infectious virus from 1–2 days after inoculation (10^7^TCID50/ml, high dose of inoculation), and kept shedding virus until day 7 [33]. Thus, the conditions in slaughterhouses with camels from different sources being kept from several days to weeks before being slaughtered, provide optimal conditions for virus cross-transmission and for amplification of virus infection and explains the greater virus genetic heterogeneity observed in the samples collected there.

Nineteen (76%) of the 25 full genome sequences obtained in the present study had genetic insertions, deletions or frame-shift mutations. Previous studies had reported nucleotide deletions or insertions in ORF3, ORF5 and ORF4b accessory genes in African camel MERS-CoV [13,23,28]. Thus, Ethiopian camel-originated MERS-CoV appears to demonstrate even greater genetic instability with the detection of various nucleotide indels in nsp3, ORF3, ORF5 and N genes, but not in ORF4b gene as was observed in West Africa. In the sequences obtained from the present study we confirmed that the observed indels were not minor subpopulations within the intra-host genetic diversity by variant-calling analysis and Sanger sequencing. Furthermore, we have virus isolates from four of these specimens and the virus isolates had the same genetic deletions observed in the original specimen (unpublished data) indicating the viability of these viruses. In contrast, such frequent and diverse nucleotide indels have rarely been seen in clade B strains which are enzootic in camels in the Arabian Peninsula.

Given the role of nsp3, ORF3, ORF5 and N protein in the virus replication cycle, it is important to explore the impact of indels observed in those genes of the Ethiopian camel-originated MERS-CoV on the virus phenotype, to further understand its zoonotic risk. Nsp3 protein of MERS-CoV is important for virus replication through inducing the formation of double-membrane vesicles (DMV) [34]. It encodes several domains, including papain-like protease (PLpro) which can proteolytically cleave the junction from nsp1 to nsp4 and act as an interferon antagonist [35,36]. However, the single amino acid deletion found in Ethiopian and Nigerian camel-originated MERS-CoVs is not in the PLpro domain and the implication of this deletion is unknown to date. The diversity of indel patterns in ORF3 and ORF5 gene in Ethiopian camel-originated MERS-CoV is of interest. An *in vitro* study demonstrated that ORF3 protein of MERS-CoV is able to promote cell apoptosis, similar with the ORF3a protein of SARS-CoV and SARS-CoV-2 [37–39]. The deletions of accessory gene ORF3-5 (ORF3, −4a, −4b, and −5) have been shown to attenuate the virus replication competence and increase the INF- β and IFN- λ response and ORF5 likely modulates NF-kB-mediated inflammation [40]. Still, the implication of these indel patterns of ORF3 and ORF5 genes on viral phenotype remains unknown, unless functional studies are carried out.

The deletions observed in N protein in Ethiopian camel-originated MERS-CoV are also interesting. N protein of coronaviruses packages the viral RNA to form a nucleocapsid and involves in other functions including virus replication and evasion of the innate immune response [41–43]. MERS-CoV N protein can also act as an IFN antagonist, inhibiting type I and type III interferon induction by targeting RIG-I signalling [44]. In the host cell life cycle, MERS-CoV N protein interacts with human translation elongation factor 1A(EF1A) to inhibit cytokinesis and cell proliferation [45]. These findings imply that N protein may play a role in pathogenesis. Therefore, it is of interest to explore the functional significance of the deletions found in MERS-CoV. The implication of genetic instability of MERS-CoV in camels in East Africa warrants more investigation and functional characterization is needed.

Unlike West African strains (Nigeria and Burkina Faso) with large ORF4b deletions, no ORF4b gene deletions were observed in our sequences from Ethiopia. Phenotypic characterization showed that all African clade C viruses studied, whether with (West African strains from Nigeria and Burkina Faso) or without large ORF4b deletions (some East African strains from Ethiopia and Kenya) had reduced replication competence in a human airway Calu-3 cell line, in *ex vivo* cultures of human lung and bronchus, and in the lungs of an *in vivo* mouse model, when compared to clade A and B viruses from Saudi Arabia [13,14]. Since the Ethiopian and Kenyan viruses we studied phenotypically had no ORF4b deletions, the difference in observed phenotype was not primarily determined by the ORF4b deletions. Similar studies are needed to phenotypically characterize the newly observed genetic indels in some of the Ethiopian viruses detected in this study.

Overall, the frequency and diversity of genome indels observed in African clade C viruses reported in this study indicates the virus genetic instability and raises the possibility that MERS-CoV is a virus that is still adapting to its ecologic niche in this region. There is little surveillance or virus genetic data from regions of Central Asia, East of the Arabian Peninsula and such information is important to assess whether this genetic instability is also observed outside the African Continent.

This study has contributed to the existing knowledge on the transmission dynamics and the genetic characteristics of MERS-CoV in dromedary camels in Ethiopia and Eastern Africa. The frequency and diversity of nucleotide insertions and deletions observed in African MERS-CoV suggesting genetic instability of MERS-CoV in camels raise the concern about the possibility of the emergence of novel viruses with unexpected transmissibility and pathogenicity for humans. A deletion in ORF8 gene was associated with the SARS-CoV-1 acquiring efficient transmission in humans[46]. The high camel density and high prevalence of MERS-CoV infection in camels in East Africa, evidence of human infection in Africa and the observed genetic instability of MERS-CoV in East Africa strongly suggest that further surveillance of MERS-CoV in camels and humans in Africa is critically needed in order to better understand the zoonotic risk and monitor the virus evolution.

## Supplementary Material

Supplemental_material.docx
