# Supplementary material for: Genetic diversity and molecular epidemiology of Middle East Respiratory Syndrome Coronavirus in dromedaries in Ethiopia, 2017–2020
Source: Emerg Microbes Infect. 2023 Jan 27;12(1):e2164218. doi: 10.1080/22221751.2022.2164218 (PMC9888459; doi:10.1080/22221751.2022.2164218)
Supplement: Supplemental_material.docx [file TEMI_A_2164218_SM6970.docx]

Table S1. Sequences used for phylogenetic analysis as shown in Fig.2. Reference sequences (n=75) in clades A, B, and C of MERS-CoV were downloaded from Genbank and those highlighted in red are the whole genome sequences (n=25) obtained from this Ethiopia surveillance study. Among them, sequences of MZ268404 (CAC9690), MG923466 (HKU4412), MG923467 (HKU4448) and MG923468 (HKU4458) were published before[1,2].

| **Sample NO.** | **Sample type** | **Collection place** | **Type of collection place** | **Collection time** | **Herd owner** |
| --- | --- | --- | --- | --- | --- |
| CAC4366 | Nasal swab | Amibara | Herd | 2017-01-12 | A-1 |
| HKU4412 | Nasal swab | Amibara | Herd | 2017-01-13 | A-2 |
| HKU4448 | Nasal swab | Amibara | Herd | 2017-01-13 | A-3 |
| HKU4458 | Nasal swab | Amibara | Herd | 2017-01-13 | A-3 |
| HKU4459 | Nasal swab | Amibara | Herd | 2017-01-18 | A-4 |
| CAC4749 | Nasal swab | Chifra | Herd | 2017-12-07 | C-1 |
| CAC4752 | Nasal swab | Chifra | Herd | 2017-12-07 | C-2 |
| CAC4753 | Nasal swab | Chifra | Herd | 2017-12-07 | C-2 |
| CAC4787 | Nasal swab | Gewane | Herd | 2017-12-27 | G-1 |
| CAC4791 | Nasal swab | Gewane | Herd | 2017-12-27 | G-2 |
| CAC4802 | Nasal swab | Gewane | Herd | 2017-12-27 | G-2 |
| CAC4849 | Nasal swab | Babile | Slaughterhouse | 2017-10-11 | / |
| CAC4855 | Nasal swab | Babile | Slaughterhouse | 2017-10-09 | / |
| CAC4868 | Nasal swab | Babile | Slaughterhouse | 2017-10-11 | / |
| CAC9648 | Nasal swab | Akaki | Slaughterhouse | 2019-09-23 | / |
| CAC9650 | Nasal swab | Akaki | Slaughterhouse | 2019-09-23 | / |
| CAC9670 | Nasal swab | Akaki | Slaughterhouse | 2019-10-29 | / |
| CAC9690 | Nasal swab | Akaki | Slaughterhouse | 2019-11-24 | / |
| CAC9691 | Nasal swab | Akaki | Slaughterhouse | 2019-11-24 | / |
| CAC11175 | Turbinate swab | Amibara | Herd | 2019-11-19 | / |
| CAC11179 | Turbinate swab | Amibara | Herd | 2019-11-19 | / |
| CAC11181 | Turbinate swab | Amibara | Herd | 2019-11-20 | / |
| CAC11188 | Turbinate swab | Amibara | Herd | 2019-11-21 | / |
| CAC11191 | Turbinate swab | Amibara | Herd | 2019-11-22 | / |
| CAC11202 | Turbinate swab | Amibara | Herd | 2019-11-23 | / |

Table S2. Sample information of MERS-CoV whole genome sequences obtained in this study. Among them, CAC4849, CAC4855, and CAC4868 were slaughtered in Babile but originally from Babile, Fafan and Fafan respectively.

1. Chu DKW, Hui KPY, Perera R, et al. MERS coronaviruses from camels in Africa exhibit region-dependent genetic diversity. Proc Natl Acad Sci U S A. 2018 Mar 20;115(12):3144-3149.

2. Zhou Z, Hui KPY, So RTY, et al. Phenotypic and genetic characterization of MERS coronaviruses from Africa to understand their zoonotic potential. Proc Natl Acad Sci U S A. 2021 Jun 22;118(25).
